# Comparison of Conventional and Nonconventional Hydrogen Bond Donors in Au^–^ Complexes

**DOI:** 10.1021/acs.jpca.2c02725

**Published:** 2022-06-10

**Authors:** Jenny Triptow, Gerard Meijer, André Fielicke, Otto Dopfer, Mallory Green

**Affiliations:** † Fritz-Haber-Institut der Max-Planck Gesellschaft, Faradayweg 4-6, 14195 Berlin, Germany; ‡ Institut für Optik und Atomare Physik, Technische Universität Berlin, Hardenbergstraße 36, 10623 Berlin, Germany

## Abstract

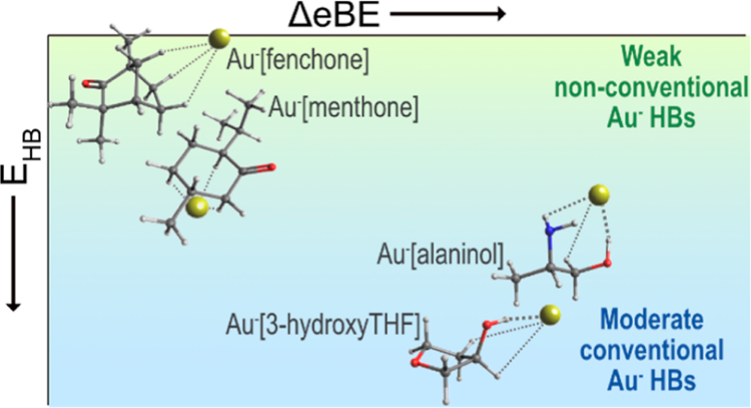

Although gold has become a well-known nonconventional hydrogen bond acceptor, interactions with nonconventional hydrogen bond donors have been largely overlooked. In order to provide a better understanding of these interactions, two conventional hydrogen bonding molecules (3-hydroxytetrahydrofuran and alaninol) and two nonconventional hydrogen bonding molecules (fenchone and menthone) were selected to form gas-phase complexes with Au^–^. The Au^–^[M] complexes were investigated using anion photoelectron spectroscopy and density functional theory. Au^–^[fenchone], Au^–^[menthone], Au^–^[3-hydroxyTHF], and Au^–^[alaninol] were found to have vertical detachment energies of 2.71 ± 0.05, 2.76 ± 0.05, 3.01 ± 0.03, and 3.02 ± 0.03 eV, respectively, which agree well with theory. The photoelectron spectra of the complexes resemble the spectrum of Au^–^ but are blueshifted due to the electron transfer from Au^–^ to M. With density functional theory, natural bond orbital analysis, and atoms-in-molecules analysis, we were able to extend our comparison of conventional and nonconventional hydrogen bonding to include geometric and electronic similarities. In Au^–^[3-hydroxyTHF] and Au^–^[alaninol], the hydrogen bonding comprised of Au^–^···HO as a strong, primary hydrogen bond, with secondary stabilization by weaker Au^–^···HN or Au^–^···HC hydrogen bonds. Interestingly, the Au^–^···HC bonds in Au^–^[fenchone] and Au^–^[menthone] can be characterized as hydrogen bonds, despite their classification as nonconventional hydrogen bond donors.

## Introduction

Historically, hydrogen bonds (H bonds) have exclusively been attributed to interactions between hydrogen and the most electronegative elements: O, N, and F. However, increasing evidence of interactions involving alternative atoms, which possess the same characteristics of conventional H bonding, has encouraged IUPAC to revisit the former restrictive definition of a H bond.^1−10^ In general, IUPAC describes a H bond as an attractive interaction in which a hydrogen that is covalently bound to an electronegative atom (HX) acts as the H bond donor and interacts with another electronegative atom, Y (with electron donor character), which is assigned as the H bond acceptor.^11^ The resulting H bond, Y···HX, is a three-center-four-electron (3c-4e) bond.^12,13^

The new, and more inclusive, definition of H bonding by IUPAC centers the focus on the geometric, energetic, and electronic properties of the interaction, as opposed to definitions reliant on the identity of atoms involved in the interaction.^11^ Such geometric properties include a Y···HX bond that approaches linearity (∠XHY = 110–180°) and a Y···H bond length that falls below the van der Waals radii of the H and Y atoms. Also, it is common to see lengthening of the HX bond upon H bonding, which leads to a redshift in the H–X stretching frequency and an increase in the infrared absorption cross-section for the H–X stretching vibration.

The forces involved in the formation of a H bond can be electrostatic, inductive, or dispersive in nature. The electrostatic component of a H bond is the dipole–dipole interaction between the donor and acceptor. Induction and dispersion forces can both be present in the formation of a H bond, but the significance of their contribution to the H bond can depend on the charge character of the proton donor and acceptor.^14^

With the extension of the definition of a H bond, many nonconventional H bond acceptors have been identified. Transition metals have been found to be particularly good H bond acceptors.^10,15−19^ As a result, Brammer has suggested that transition metals as H bond acceptors must be electron-rich with filled d shells, indicating that the late transition metals are the most effective H bond acceptors.^2^ Gold is a late transition metal with an electron configuration of [Xe]4f^14^5d^10^6s^1^. Hence, this element has many atomic properties that are advantageous for H bonding. Gold is known to be highly relativistic, which leads to contractions of the atomic and ionic radii. This contraction provides potential H bond donors greater accessibility to the atom for bonding. Another outcome of gold’s large relativistic effects is that gold is one of the most electronegative transition metals, with an electronegativity similar to that of the heavier halogens. A high electronegativity and high polarizability enable Au to behave as a Lewis base. This metallobasicity has been determined to be an asset in forming H bonds with gold.^20^ Due to gold’s high electron affinity (EA), the auride anion, Au^–^, can readily be formed. This additional negative charge can strengthen attractive forces in a H bond in what is known as an ionic or charge-assisted H bond.^21−23^

H bonding of the auride anion (Au^–^···HX) has been studied extensively with both theory and experiments. Many of the earlier studies included computational and gas-phase studies evaluating the H bonding between Au^–^ and prototypical H bond donors, such as (H_2_O)_*n*_,^24−26^ (NH_3_)_*n*_,^27,28^ and (HF)_*n*_.^29^ In these complexes, it was found that Au^–^ could sustain moderate to strong H bonding. From the prototypical examples, explorations with more complicated hydrogen-bound systems were conducted. For example, microsolvation of Au^–^ and Au_2_^–^ by polar solvents has been studied in the gas phase.^30,31^ Additionally, Cao et al. investigated H bonding between Au^–^ and nucleobases to understand the interactions between gold and DNA.^32^

These investigations of H bonding in Au^–^···HX complexes have been overwhelmingly centered on interactions between gold and conventional H bond donors: HO, HN, and HF. There are few studies which provide thorough evaluations of Au^–^···HC interactions as their contributions to the H bonding in a system can be overshadowed by the presence of stronger H bond donors. In addition, there are cases in which these interactions cannot be conclusively identified as H bonds.^5,32,33^

Despite the lack of examples of Au^–^···HC bonding in the gas phase, there is much support for the importance of this interaction in chemical systems. Specifically, the Au^–^···HC interaction is expected to be an important secondary interaction in catalysis.^33,34^ Additionally, several molecules, important in biology and chemistry, do not possess conventional H bond donors and therefore can only offer HC as a potential H bond donor. For example, fenchone (fen) and menthone (men) are both cyclic monoterpenoids, characterized by their almost fully saturated hydrocarbon structure. The only functional group present in these molecules is a ketone, which cannot act as a H bond donor. Interestingly, these molecules are members of a larger class of compounds, which have shown to be useful in the green synthesis of gold nanoparticles.^35−37^ Even though these molecules do not contain a conventional H bond-donating functional group, they have indicated potential chemical activity with gold. The study of the interaction of these molecules with gold can provide insight into the potential H bonding between gold as a nonconventional H bond acceptor and nonconventional H bond donors, such as HC.

We have studied four auride–organic complexes, utilizing a combination of velocity map imaging (VMI) spectroscopy and density functional theory (DFT) computational methods. VMI spectroscopy of size-selected anions is well-suited for the investigation of these species as the recorded photoelectron carries both energetic and angular information of the complex.^38,39^ The computational methods we have employed are commonly used for characterizing H bonding, which allows for straightforward comparisons to previously reported Au^–^···HX complexes.

The four complexes investigated can be distinguished by the presence of conventional and non-conventional H bond-donating functional groups, and are all notably chiral. The nonconventional bonding molecules include fenchone and menthone, as detailed above. The molecules designated as conventional H bond donors are 3-hydroxytetrahydrofuran (3-HTHF) and alaninol (ala). 3-HTHF contains a hydroxy group and is often used as a standard for measuring H bonding.^40,41^ Alaninol is an alcohol derivative of the amino acid, alanine, which contains two functional groups (NH_2_ and OH) that are expected to form H bonds with Au^–^. In this article, we provide spectroscopic and theoretical evidence of the interactions in the auride complexes, Au^–^[fen], Au^–^[men], Au^–^[3-HTHF], and Au^–^[ala], to provide comparisons of the specific nature of bonding in these seemingly different complexes. An understanding of these interactions can improve our knowledge of hydrogen bonding in nonconventional cases and provide the foundation needed to utilize these chemical systems in studies of photoelectron circular dichroism of chiral anions.

## Methods

### Experimental Methods

**Figure 1:**
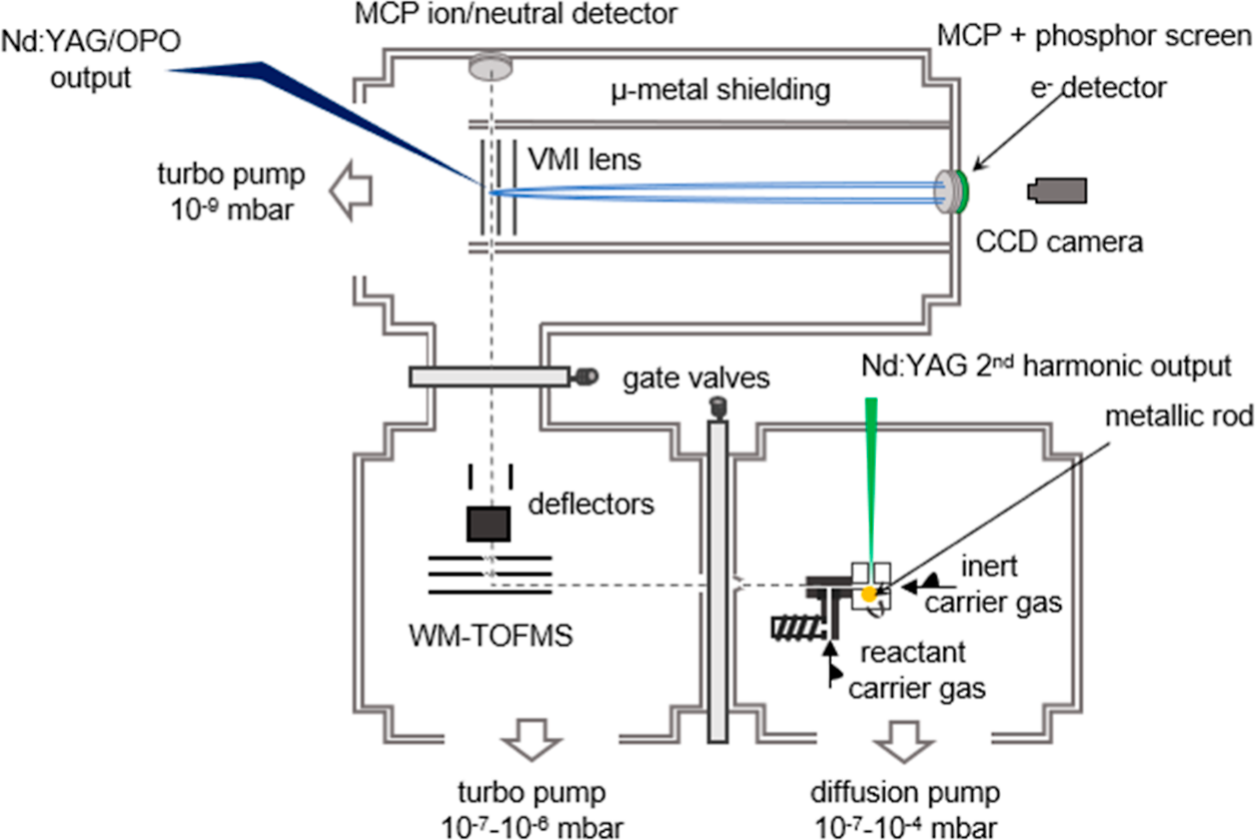
Experimental setup consisting of a laser ablation source, a linear TOF mass spectrometer, and a VMI spectrometer for the photodetachment of Au^–^[M].

A schematic of the experimental setup is shown in Figure 1, which consists of three main components: a laser ablation source for Au^–^[M] (where M = fen, men, 3-HTHF, or ala) production, a linear time-of-flight mass spectrometer (TOFMS) for *m*/*z* separation via flight times, and a VMI spectrometer for the recording of photoelectron angular distributions (PADs). The experiment runs at 10 Hz.

The chambers are separated by small apertures to enable differential pumping. The source chamber is pumped using a diffusion pump and can reach an absolute pressure of 10^–7^ mbar. During source operation, the chamber pressure rises to 10^–5^ to 10^–3^ mbar. A pressure of around 10^–6^ and 10^–8^ mbar is maintained using the turbomolecular pumps in the TOF and VMI chambers.

The laser ablation source is modeled off of the design by Smalley and Bondybey.^42−44^ The source was originally designed for the study of isolated metallic clusters, and an in-depth description is available elsewhere.^45^ Here, a short description of the implementation of the source for creation of gold–organic complexes will be provided.

The laser ablation source consists of two main parts: the ablation block, in which the production of metal ions via laser ablation occurs, and the reaction block, which introduces the organic molecules. Laser ablation is performed with the second harmonic (532 nm with 1–12 mJ/pulse) of a ns-pulsed Nd:YAG laser (Quantel Brio). The laser output is focused with an adjustable convex lens (*f* = 250 mm) on a gold rod, which is both translating and rotating, in order to provide a continually fresh surface for ablation. A pulsed Parker general valve introduces the helium carrier gas, perpendicular to the target and the laser beam, which carries the laser ablation products, among them Au^–^, to the reaction block.

The reaction block is mounted downstream to the exit channel of the ablation block. The reaction block is connected to a second valve to introduce the organic molecules, which are stored in a reservoir close to the valve. Due to their relatively high vapor pressure, the molecules are readily brought into the gas phase and seeded in the helium carrier gas. In the reaction block, the molecules react with Au^–^ to form Au^–^[M] complexes.

The molecular beam enters the TOF chamber after passing through a skimmer with a 2 mm opening. The anions are mass-separated via a perpendicular Wiley McLaren time-of-flight mass spectrometer (WM-TOFMS).^46^ Anions are extracted down the TOF axis using fast-switching electric fields on the WM-TOFMS electrodes. Typical acceleration voltages are 3–4 kV. After the TOF electrodes, two sets of deflector plates can be used to optimize the flight path of the anions. A microchannel plate detector (MCP) is placed along the TOF axis to record anion signals, in order to provide a mass spectrum. Additionally, by applying a negative voltage to a wire grid placed before the MCP, all anions can be deflected in order to observe the neutral molecules originating from the photodetachment process that occurs in the VMI region. The resolution of the mass spectrometer is approximately *m*/Δ*m* = 500.

The mass-selected complex is photodetached using an optical parametric oscillator (OPO Panther Ex, continuum λ = 2000–215 nm) system pumped by the third harmonic (355 nm) of a Surelite II Nd:YAG laser, at a photodetachment energy of either 4.13 or 4.35 eV. The incident photon beam intercepts the anion beam, perpendicularly, and is synchronized to the time of arrival of Au^–^[M] in the VMI interaction region.

Photodetached electrons are measured with a VMI spectrometer that is oriented approximately perpendicular to the TOF axis. As the photoelectrons carry an additional velocity component due to the trajectory of their parent molecule, the spectrometer is placed at a slight angle off normal to compensate for this additional velocity. The VMI optical design is based on the setup of Eppink and Parker^47^ and is encased in a double-μ-metal shield, to prevent magnetic distortions of the photoelectron trajectory. The imaging detector consists of a set of two MCPs in a chevron arrangement, coupled to a P47 phosphor screen and a CCD camera. The current configuration of the VMI spectrometer enables acquisition of a large range (up to 4 eV) of electron kinetic energies (eKEs) and the full PAD. The VMI spectrometer can achieve a resolution of ΔeKE/eKE = 3.4%. The data collection is performed with a custom LabView program, which allows for raw and centroid image accumulation. The accumulated photoelectron images (accumulated over >50,000 experimental cycles) are reconstructed using the maximum entropy velocity Legendre reconstruction (MEVELER) method to provide a final photoelectron spectrum and reconstructed photoelectron distribution.^48^ The VMI spectrometer is calibrated with Au^–^, using the precisely known EA and excitation energies of the gold atom.^49,50^

### Computational Methods

DFT calculations of the Au^–^[M] complexes have been carried out with the hybrid density functional B3LYP^51−53^ and the D3 dispersion correction of Grimme (B3LYP-D3).^54^ The augmented correlation-consistent polarized valence-only triple-zeta basis set (aug-cc-pVTZ) is used for H, C, N, and O.^55−59^ For Au, a pseudopotential is added (aug-cc-pVTZ-PP).^60,61^ All calculations are performed with the GAUSSIAN16 software package.^62^

To build the complex, geometries of the bare organic molecules are optimized first. Stable starting geometries of conformers of the four organic molecules have been acquired from previous studies.^63−66^ Overall, one conformer of fen, four conformers of men, two conformers of 3-HTHF, and five conformers of ala were optimized to provide a base for constructing the complex. Complexes were constructed by placing Au^–^ at six different positions around the neutral molecule. After placing Au^–^ at a unique position, geometry optimizations were carried out on the anionic complex. Additionally, harmonic frequency calculations were performed to ensure that the geometry of the complex has reached a minimum. Harmonic frequencies were scaled by a constant factor of 0.9636, determined from the ratio of the B3LYP-D3/aug-cc-pVTZ-calculated frequencies of the symmetric and antisymmetric stretch modes of H_2_O and their corresponding experimental values.^67^ From these calculations, vertical detachment energies (VDEs), anionic dissociation energies (*D*_0_^–^), and Au^–^ binding energies (BE_Au_ = −*D*_0_^–^) are determined. All energies contain zero-point energy corrections.

The molecular orbital structures were analyzed for the lowest-energy complex isomers. Natural bond orbital (NBO) analysis of the complexes was performed to determine the charge distribution in Au^–^[M] and Au[M] and to provide understanding of the electron donation character from Au^–^ to the organic molecule. The resulting molecular orbitals and partial charge distributions were visualized using the open-source molecular builder and visualization tool, Avogadro 1.2.0.^68^

To obtain a better understanding of the strength of H bonding of the studied complexes, Bader’s quantum theory of atoms in molecules (QTAIM)^69^ is employed through the Multiwfn code and visualization tool (version 3.8).^70^ The optimized wavefunctions of the anionic complexes are used in order to determine bond critical points (BCPs) and BCP electron densities (ρ_BCP_) of the H bonding interactions.

## Results & Discussion

### Experimental Results

**Figure 2:**
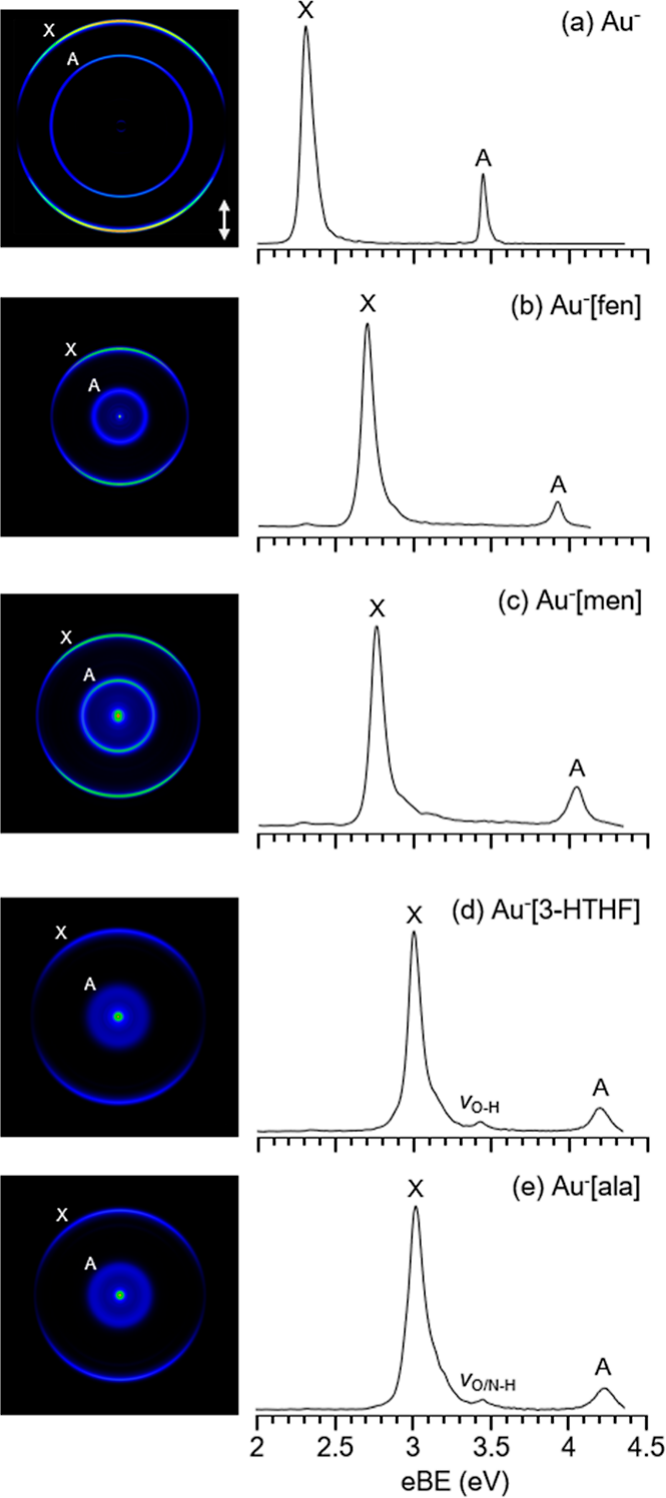
Reconstructed photoelectron distributions and photoelectron spectra of (a) Au^–^, (b) Au^–^[fen], (c) Au^–^[men], (d) Au^–^[3-HTHF], and (e) Au^–^[ala], taken at photon energies of 4.35 eV (a,c–e) and 4.13 eV (b). The double arrow indicates the polarization direction of the photodetachment laser.

The reconstructed photoelectron distributions and the radially integrated photoelectron spectra of Au^–^ and the four auride complexes (Au^–^[fen], Au^–^[men], Au^–^[3-HTHF], and Au^–^[ala]) can be found in Figure 2. The spectrum of Au^–^ reveals two atomic transitions. The transition at lower electron binding energies (eBEs), labeled X in the spectra, is the anionic ground state (^1^S_0_) to neutral ground state (^2^S_1/2_) transition, which corresponds detachment of the electron from the 6s orbital. The center of this peak occurs at 2.3086 eV, which is the EA of Au.^49^ The transition at higher eBE, labeled A, is the transition to the first excited state of the neutral atom [Au^–^ (^1^S_0_) → Au (^2^D_5/2_)] and corresponds to electron detachment from the 5d orbital. This peak is centered at 3.4445 eV.^49,50^ Comparing the spectra of the complexes to those of Au^–^, it is clear that all complex spectra carry photodetachment transitions that are similar to the atomic transitions of Au^–^ but slightly broadened and shifted to higher binding energies (see Table 1). The slight broadening is due to unresolved rotational and vibrational structures. The blue-shifted energies of the transitions indicate that the photodetachment process in the complexes can be described as electron detachment from an atom, perturbed by the attractive interactions with the complexing molecule.^26^ Therefore, the labeling of transitions in the Au^–^[M] spectra mirrors that of bare Au^–^. Such complexation-induced blueshifts in the binding energies (ΔeBE) of transitions have been observed for other Au^–^···HX complexes.^24,26,30−32^ For all complexes, there is a greater shift for A compared to X. An explanation for this can be found in the section labeled “H bond character”.

**Table 1:** Experimental eBEs and eBE Shifts From Gold Atom Detachment (Δ_eBE_), Given in eV for Au^–^ and Au^–^[M]

	eBE		ΔeBE	
species	X	A	X	A
Au^–^	2.3086^a^^,^^b^	3.4445^a^^,^^b^		
Au^–^[fen]	2.71 ± 0.05	3.93 ± 0.02	0.40	0.49
Au^–^[men]	2.76 ± 0.05	4.04 ± 0.02	0.45	0.60
Au^–^[3-HTHF]	3.01 ± 0.03	4.22 ± 0.01	0.70	0.77
Au^–^[ala]	3.02 ± 0.03	4.23 ± 0.01	0.71	0.78

a Experimentally verified transition energies of Au^–^ from refs (49) and (50).

b The FWHM of the Au^–^ X and A peaks are 0.09 and 0.04 eV.

The use of VMI spectroscopy enables analysis of the PAD, which can further the understanding of the anionic orbital character of the photodetaching electron. The reconstructed photoelectron distributions of Au^–^ and Au^–^[M] exhibit two prominent rings, whose radii correspond to the eKE of the detaching electron associated with the two main transitions in the spectra. In the image of Au^–^, there is a clear anisotropy in the photoelectron signal of the X transition in the direction of the laser polarization. The anisotropy parameter of electron detachment (β) is given using the Cooper–Zare formula,^71^ which is defined by the angular momentum of the parent orbital (*l*) and the free-electron partial waves, with angular momentum *l*_e_ = *l* ± 1. Within this formulation, there is a β dependence on the eKE of the outgoing electron. However, for the specific case of detachment of an electron from an s atomic orbital, the anisotropy parameter is calculated to be at a maximum for all eKEs, β = 2.^72^ Without the dependence on kinetic energy, a straightforward analysis of the X transition can be made for Au^–^ and the complexes. In Table 2, the value of β determined for the X transition of Au^–^ reaffirms detachment of the electron from the 6s orbital of gold. When Au^–^ forms a complex, orbitals of the complexing molecule mix with the 6s orbital of Au^–^. Contributions of orbitals with different angular momenta alter the β value and introduce an eKE dependency.^72^ In Table 2, the effect on β is apparent in the experimental anisotropy of the complexes. All complexes are found to have similar deviations in the anisotropy at similar eKEs, in comparison to Au^–^. This result supports the description of the complex spectra as perturbed atomic detachment of the Au^–^. The anisotropy parameters for the A transition signify a more isotropic detachment for all systems with β being close to 0 (Table S1).

**Table 2:** Measured Anisotropy Parameter β for the X State of the Au^–^[M] Complexes and Au^–^a

species	eKE	β_X_
Au^–^	1.82–2.04	2.00 ± 0.04
Au^–^[fen]	1.43	1.5 ± 0.2
Au^–^[men]	1.32	1.4 ± 0.3
Au^–^[3-HTHF]	1.34	1.5 ± 0.1
Au^–^[ala]	1.33	1.5 ± 0.1

a The corresponding electron kinetic energy eKE is given in eV.

The Au^–^[M] spectra with M = fen and men are most similar to the spectrum of Au^–^, indicating a smaller perturbation by the complexing molecule. For fen, the center of the X peak is 2.71 eV and the A peak is centered at 3.93 eV. The X and A peaks of Au^–^[men] are centered at 2.76 and 4.04 eV, respectively. The center of the X peak in the Au^–^[M] spectra is taken to be the VDE of the anionic complex. The shifts in energy of these two complexes are listed in Table 1. Fen exhibits a smaller shift than men for both its X and A peaks, and both complexes exhibit smaller shifts compared to Au^–^[3-HTHF] and Au^–^[ala]. Currently, there are no reported gas-phase studies of Au^–^···HC interactions for comparison. However, predictions of the VDE of Au^–^[CH_4_] indicate a much smaller expected shift in VDE (ΔeBE_X_ = 0.047 eV), in comparison to what has been observed for the Au^–^[fen] and Au^–^[men].^5^ Au^–^[men] exhibits similar shifts in VDE to Au^–^[CH_3_SH] (ΔeBE_X_ = 0.42 eV) and Au^–^[H_2_O] (ΔeBE_X_ = 0.45 eV).^26,73^ In both spectra, there is a small peak centered at 2.31 eV, which has been attributed to photodetachment of background Au^–^ caused by dissociation of the complexes during acceleration.

The eBEs for Au^–^[3-HTHF] and Au^–^[ala] are higher in energy compared to the other two complexes, indicating a greater perturbation by 3-HTHF and ala on Au^–^, compared to fen and men. Au^–^[3-HTHF] and Au^–^[ala] show larger shifts in VDE compared to other Au^–^···HO interacting complexes, such as Au^–^[H_2_O] and Au^–^[CH_3_OH] (ΔeBE_X_ = 0.51 eV).^26,73^ However, the interactions of Au^–^ with nucleobases, studied by Cao et al., produced much greater VDE shifts (ΔeBE_X_ = 0.93–1.13 eV).^32^

In addition to the two main features that are apparent for all the complexes, there is a third feature that manifests in the spectra of Au^–^[3-HTHF] and Au^–^[ala]. This feature is centered at 3.43 eV in Au^–^[3-HTHF] and at 3.45 eV in Au^–^[ala]. It occurs 3410 cm^–1^ (0.42 ± 0.04 eV) above the ground-state transition in Au^–^[3-HTHF] and 3440 cm^–1^ (0.43 ± 0.03 eV) above the ground-state transition in Au^–^[ala] (Table 3).There are two possible assignments that can be made for this peak: (1) The energy of this peak in both spectra is rather close to the eBE_A_ of Au^–^. As the Au^–^[fen] and Au^–^[men] spectra exhibited a signal that was attributed to the X transition of background Au^–^, it is possible for this peak to be attributed to the A transition of background Au^–^. However, the Au^–^[3-HTHF] and Au^–^[ala] spectra do not exhibit an additional Au^–^ X transition with a relative intensity that would support this assignment. (2) The proximity of this new feature to the ground-state transition in both spectra (∼3400 cm^–1^) is in line with what would be expected for activation of the OH stretching mode of the molecule upon photodetachment (Table 3). Additionally, this assignment is supported by the fact that similar vibrational features were observed in the photodetachment spectrum of Au^–^[H_2_O].^26^ As such, we favor this assignment of the spectral feature and have, therefore, labeled it as ν_O–H_ in the Au^–^[3-HTHF] spectrum. In Au^–^[ala], the frequency of the stretching of the −NH_2_ group also falls within the confidence interval of this feature (see Table S2). As the Au^–^···HN interaction can be the primary attractive force for some of the higher-energy complex isomers (see section Structural Analysis: Au^–^[3-HTHF] and Au^–^[ala]), the feature in the Au^–^[ala] spectrum is labeled “ν_O/N–H_” to account for the different possible contributions.

**Table 3:** Comparison of the Experimental ν_O–H_ in Au^–^[3-HTHF] and ν_O/N–H_ Au^–^[ala] with the Theoretically Scaled ν_OH_ of the Au-Bound Complex (T1.1 and A1.1) and Isolated Molecule (T1 and A1)^a^

species	exp.	Au[M]	Au[M]	M
Au^–^[3-HTHF]	3410 ± 330	3107	3628	3643
Au^–^[ala]	3440 ± 260	3145	3213	3583

a Frequencies are given in cm^–1^.

### Computational Results

#### Structural Analysis

We have calculated 4 isomers of Au^–^[fen], 11 isomers of Au^–^[men], 6 isomers of Au^–^[3-HTHF], and 15 isomers of Au^–^[ala]. A subset of low-energy isomers for each complex has been selected for presenting here to demonstrate the common structural and binding characteristics of each complex. Structures of the Au^–^[M] isomers and additional geometry details not discussed in the text can be found in the Supporting Information (Figures S1–S3 and Tables S3–S6).

The molecules M = fen, men, 3-HTHF, and ala have been assigned the tags F, M, T, and A, respectively. The isolated molecules are listed in order of increasing relative energy (i.e., lowest-energy isomer of ala is A1), and the complexes are named according to the organic isomer used to construct the complex and the complex energy relative to the lowest energy complex isomer in the set (i.e., the lowest-energy complex formed with the lowest-energy conformer of ala would be named A1.1).

#### Au^–^[fen] and Au^–^[men]

**Figure 3:**
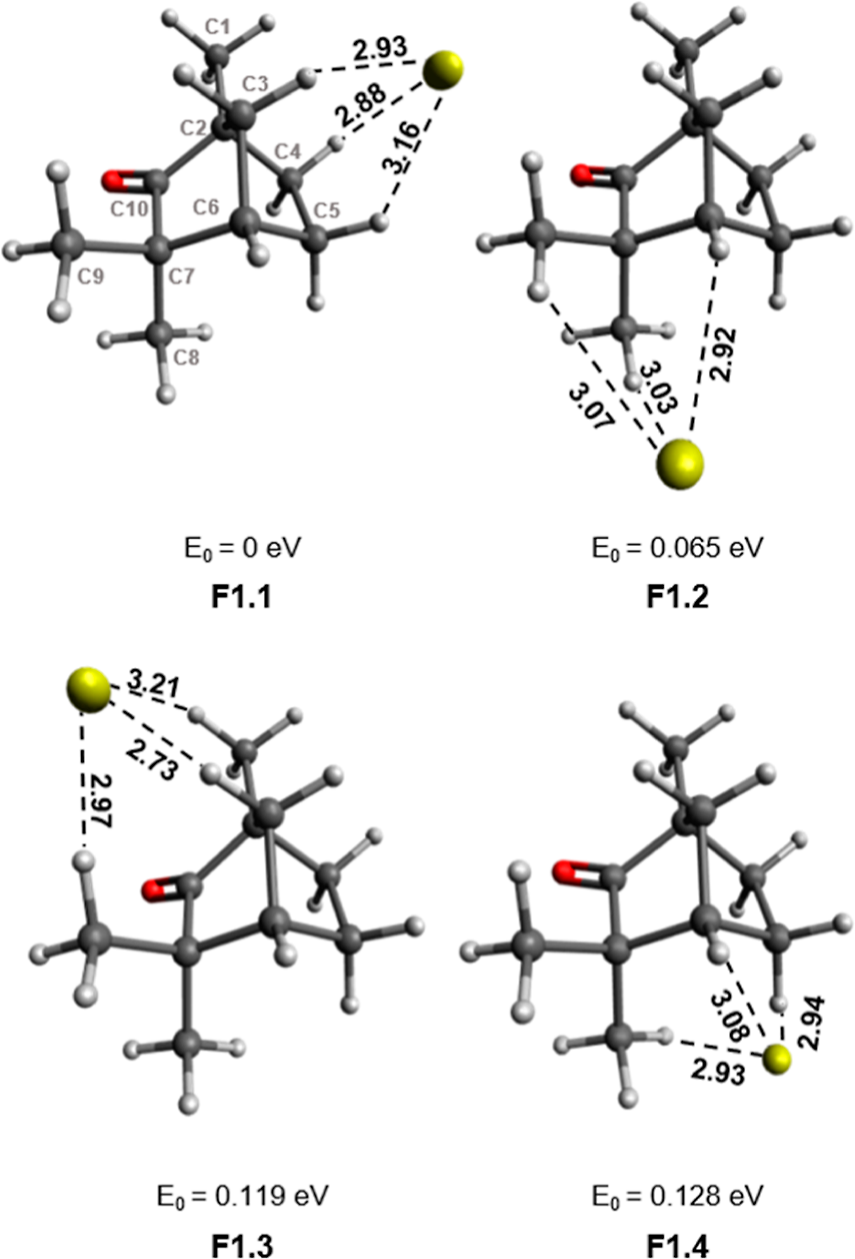
Isomers of Au^–^[fen]. Bond lengths are given in Å, and the relative energies listed are given with respect to the lowest-energy complex. The labeling of atoms in F1.1 applies to all complexes.

As evident from Figure 3, fen has a rigid structure, and only one conformer is possible.^65^ From the single conformation of fen, four optimized complexes could be calculated, as seen in Figure 3. For each complex, the gold atom’s relative position is marked by dotted lines to the three closest hydrogen atoms of the molecule. The energy (*E*_0_) given is the energy relative to the lowest-energy complex (*E*_0_ = 0 eV). The latter has Au^–^ stabilized by one of the five-membered rings of fen, which is oriented opposite of the carbonyl group (C10–O), to reduce repulsion between the electronegative O and Au^–^. All four Au^–^[fen] isomers indicated an avoidance of the CO group. The F1.3 isomer is similar to F1.1, in that Au^–^ is stabilized by the other side of the bicyclic structure. However, it is higher in relative energy due to its proximity to the CO group. Although the position of Au^–^ in F1.2 is furthest from CO, the average Au^–^···H distances are longer, indicating a weaker interaction.

Upon interaction of fen with Au^–^, there is no significant lengthening of the CH bonds. For all the isomers characterized, the Au^–^···H distances are between 2.73 and 3.21 Å and the bond angle ∠AuHC ranges from 128.4 to 168.4°. The smallest Au^–^···H distances and ∠AuHC closest to linearity are present in the F3 isomer. As this is not the lowest-energy isomer, it is clear that the attraction between Au^–^ and fen is not derived from a solitary Au^–^···HC interaction.

**Figure 4:**
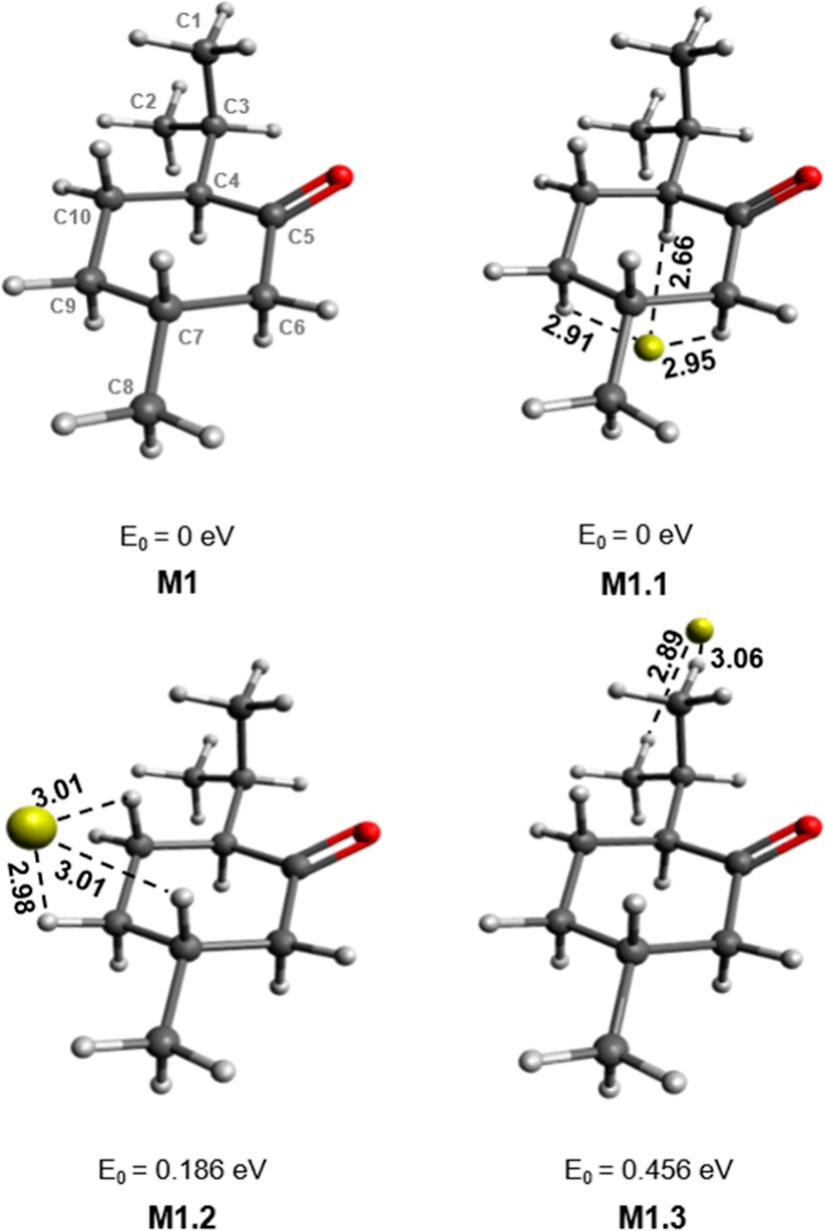
Menthone conformer (M1) and isomers of Au^–^[M1]. Bond lengths are given in Å and the relative energies listed are given with respect to the lowest-energy complex. The labeling of atoms in M1 applies to all complexes.

**Figure 5:**
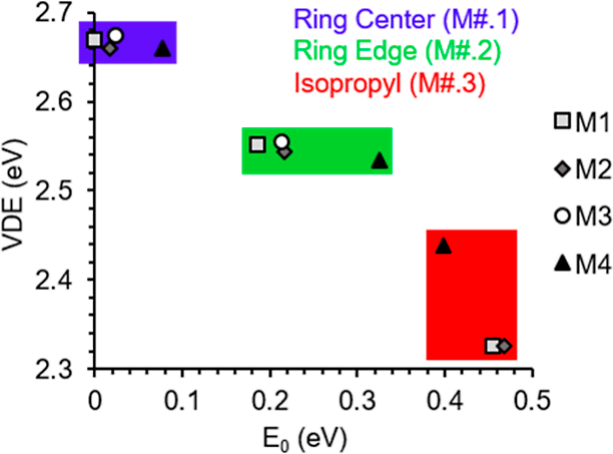
VDE vs the relative energy (*E*_0_) for all stable Au^–^[men] complexes. The graph is color-coded by the region of the men isomer that interacts with Au^–^.

Menthone is a much more flexible molecule compared to fen, and as a result, more conformers are possible. The three most stable conformers have a flat structure, with the methyl group (C8 in Figure 4) in the plane of the ring. The conformers differ from each other by a rotation of the isopropyl group (C3–C4 axis). The fourth structure has the methyl group oriented out of the plane of the ring and is the least stable configuration (see Figure S1). As the binding of Au^–^ to the different conformers of menthone indicated a similar bonding behavior, Figure 4 shows only the complexes of the lowest-energy conformer of men (M1). Similar to the Au^–^[fen] complexes, all complexes with men appear to maximize separation between Au^–^ and the partially negative O of the CO group. Binding of Au^–^ to men can be divided into three bonding motifs. The lowest-energy binding orientation is demonstrated in M1.1, in which Au^–^ is centered below the central ring. The binding of Au^–^ to the edge of the ring leads to the second set of stable Au^–^[men] complexes, as demonstrated by M1.2. The least stable isomers show a bond between Au^–^ and the isopropyl group, like is shown for M1.3. It is interesting to note that although typically the relative energy of the complex in a given orientation follows the ordering of their parent molecules, the order of relative energies for the isopropyl-bound complexes is switched, such that M4.3 has the lowest relative energy, as shown in Figure 5. This is due to additional interaction of the methyl group (see M4.3 in Figure S1). In the complexes, the CH bond lengths of the interacting hydrogen atoms undergo minor lengthening upon binding of the Au^–^. The Au^–^···H distance ranges from 2.66 to 3.08 Å and the bond angle ∠AuHC ranges from 117.7 to 161.0°, which is comparable to Au^–^ bonding in Au^–^[fen].

#### Au^–^[3-HTHF] and Au^–^[ala]

**Figure 6:**
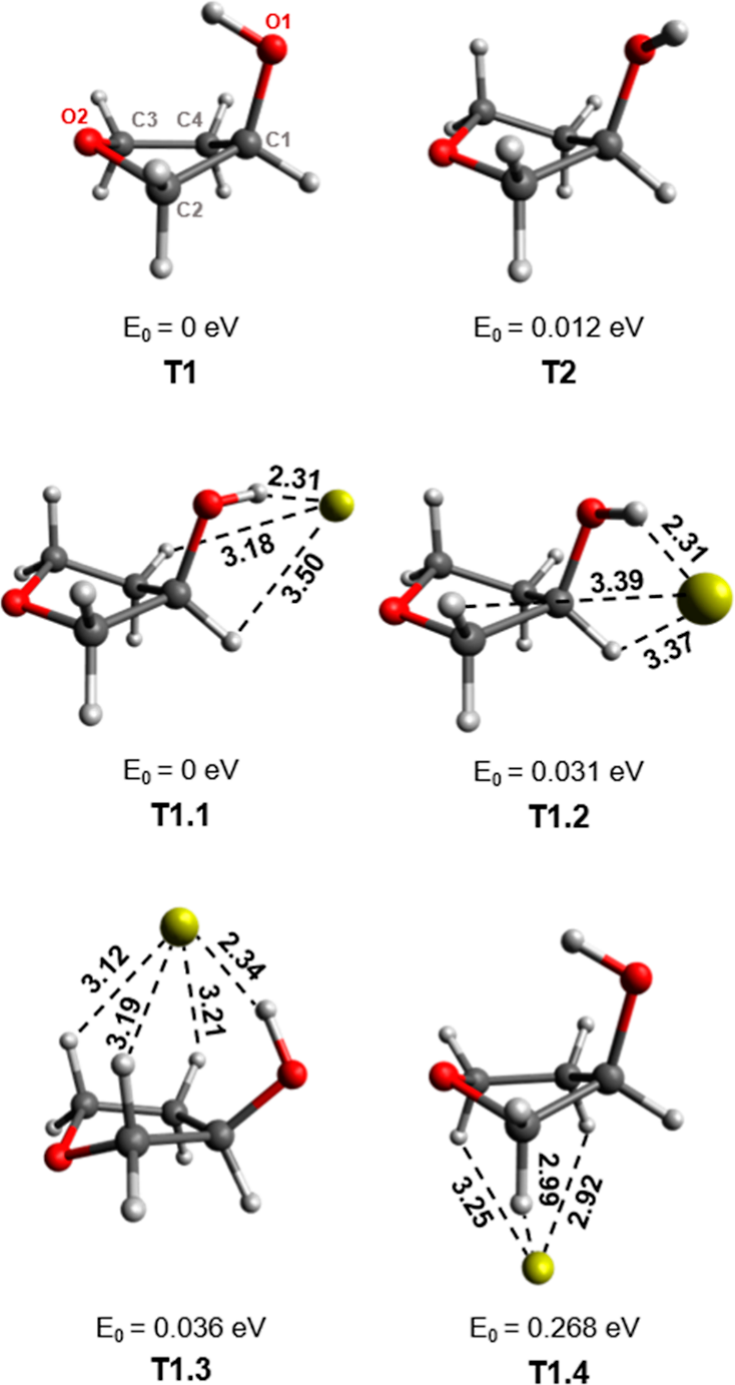
3-HTHF conformers (T1 and T2) and isomers of Au^–^[T1]. Bond lengths are given in Å and the relative energies listed are given with respect to the lowest-energy complex. The labeling of atoms in T1 applies to all complexes.

The tetrahydrofuran ring in 3-HTHF can undergo ring puckering to form different conformers. In this study, we calculated two conformers of 3-HTHF (T1 and T2) shown in Figure 6. The lower-energy conformer T1 has a C2-endo structure, where the OH group is oriented toward the center of the ring and stabilized by the internal interaction of the OH group and the ether oxygen (O2). This binding motif has been found to be the lowest-energy conformer of 3-HTHF in other studies.^64^ The second conformer, T2, exhibits a C4-endo structure, where the OH group is oriented away from the ring center. This conformer corresponds to the most predominant furanose ring structure found in nucleotides.^64^

Of the six stable Au^–^[3-HTHF] complexes found for the T1 and T2 conformers, the four lowest-energy complexes are presented in Figure 6. The lowest-energy complexes, T1.1 and T1.2, have Au^–^ positioned outside of the ring, with a strong interaction with the OH group. Although the 3-HTHF unit of these complexes bears greater resemblance to the T2 isomer, they are labeled as having a T1 starting configuration as it was found that both T1 and T2 complexes optimize to these geometries. This indicates a low barrier for conversion between these two conformers, which is supported by their minimal energy difference (0.012 eV). In T1.3, the Au^–^ position is above the ring. There is still an interaction between Au^–^ and the OH group (O1H), but it is weaker, given the longer Au^–^···H bond length, compared to T1.1 and T1.2. Accommodation of Au^–^ along the center axis of the ring indicates a puckering of the ether oxygen (O2) away from gold (T1.3 and T1.4). This aversion is analogous to the behavior between the carbonyl oxygen and Au^–^ in Au^–^[men] and Au^–^[fen]. The final isomer (T1.4) is unique in that it exhibits no interaction between Au^–^ and the OH group. Instead, Au^–^ binds to the CH groups of the ring. The significance of the Au^–^···HO interaction in the stability of the complex is evident from the large increase in relative energy compared to that of the three lower-energy complexes. The difference in Au^–^ bond strength of OH and CH in 3-HTHF is also evident from the different geometries of these bonds. For Au^–^···HO bonds, the average increase in OH bond length upon complexation is 0.03 Å, compared to an average CH increase of 0.002 Å in Au^–^···HC bonds. The Au^–^···HO bonds have much shorter Au^–^···H bond lengths (2.31–2.34 Å) and ∠AuHO bond angles closer to linearity (162.1–169.0°), in comparison to Au^–^···HC bonds (Au^–^···H = 2.99–3.50 Å and ∠AuHC = 92.2–139.5°).

**Figure 7:**
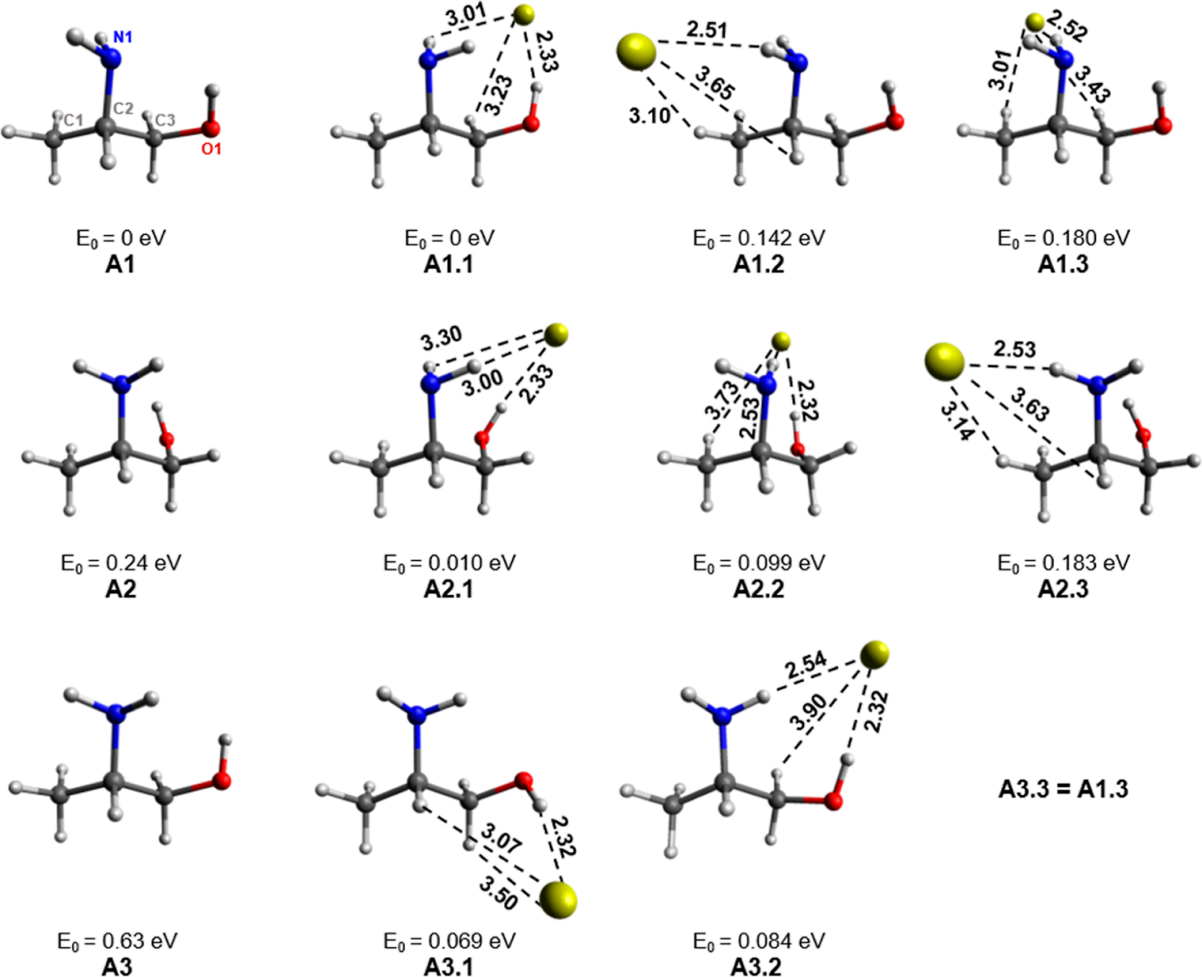
Ala conformers (A1–A3) and isomers of Au^–^[A1–A3]. Bond lengths are given in Å and the relative energies listed are given with respect to the lowest-energy complex. The labeling of atoms in A1 applies to all complexes.

Ala is another flexible molecule, which has been shown to have up to 25 conformers within a relative energy range of only 0.3 eV.^63^ Here, five conformers of ala were selected for the study, with the focus on rotations about the N1–C2, C2–C3, and C3–O1 axes. Of the five initial ala conformers, the three lowest-energy conformers and their complexes are shown (Figure 7). A1 and A2 exhibit an internal H bond between the NH_2_ and OH groups, whereby OH acts as the H bond donor (N···HO). A strong interaction between these groups lowers the energy of the molecule. The A3 isomer is an example of a conformer where this internal H bond is significantly weakened.

The relative energies of all complexes are given with respect to the lowest-energy complex, A1.1. The complexes shown indicate possible interactions of Au^–^ with OH, NH, and CH. The relative energy of a given complex can depend on the initial organic structure (if the internal H bond is maintained) but the identity of the primary H bond donors plays a much more significant role. Au^–^ can typically interact with two–three H atoms, and these interactions are classified as primary interactions based on the strength of the interaction, as indicated by the Au^–^···H bond length and ∠AuHX bond angle. The complexes that include an Au^–^···HO interaction (A1.1, A2.1, A2.2, A3.1, and A.3.2) have lower relative energies (0–0.099 eV) compared to the complexes without this interaction. The strong interaction between OH and Au^–^ is evident from the short Au^–^···H bond lengths (2.32–2.36 Å), almost linear ∠AuHO bond angles (159–175°), and significant lengthening of the OH bond from 0.96/0.97 Å in the monomer to 0.99 Å in the complex. It can be assumed that any complex with an Au^–^···HO interaction is dominated by this primary interaction. There are stable complexes without such an interaction (A1.2, A1.3, A2.3, and A3.3), but their relative energies are much higher (0.142–0.376 eV). In these complexes, Au^–^···HN is the primary interaction and the NH bond lengthens by 0.02 Å upon forming the complex. The resulting Au^–^···H bond ranges from 2.51–2.73 Å, and the resulting bond angle ranges from 150.8–167.3°.

**Figure 8:**
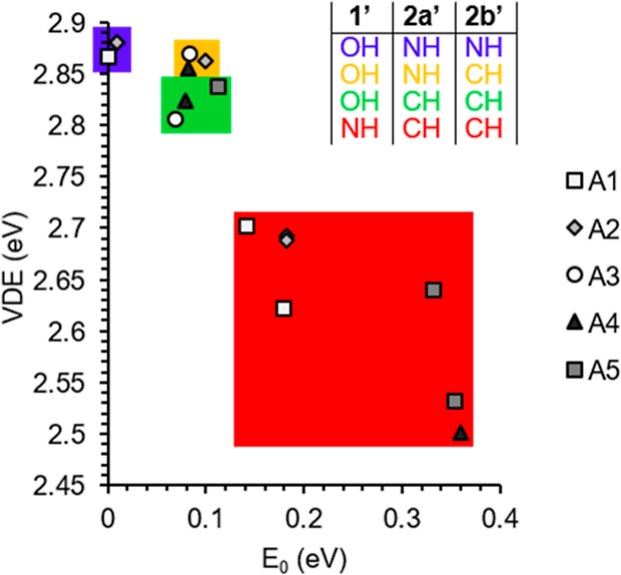
VDE vs relative energy (*E*_0_) of the Au^–^[ala] complexes. The graph is color-coded based on the primary (1′) and secondary H bond interactions (2a′ and 2b′), as indicated by the inset table.

The specific role of secondary interactions has a less straightforward, but non-negligible, effect on the stability of the complex. Figure 8 presents a graph of the VDEs of the Au^–^[ala] complexes versus their relative energies. The complexes are color-coded based on their primary (1′) and secondary (2a′/2b′) interactions. Classification as 1′, 2a′, or 2b′ is determined from the geometric properties of the interaction, which are assumed to correlate with the strength of the interaction. Therefore, the three interactions for each complex are ordered from 1′ interactions having the shortest Au^–^···H contacts and most linear ∠AuHX bond angles to 2b′ interactions, which have the longest bond lengths and the most acute angles. On comparing the relative energy of the NH- and CH-bound species, NH as a 2b′ interaction (i.e., compare blue with yellow) significantly improves the interaction with Au^–^, but a 2a′ NH interaction (compare yellow with green) does not indicate an improvement in binding of Au^–^.

### Comparing Theory and Experiments

Table 4 provides predicted relative energies (*E*_0_), anionic dissociation energies (*D*_0_^–^), VDEs, and electron binding energies of peak A (eBE_A_) of the selected complexes and Au^–^. Also, it includes the experimental VDEs and eBE_A_, for comparison. Predictions of the eBE_A_ of the different complexes are conducted utilizing the calculated molecular orbital energies and Koopman’s theorem [i.e., eBE_A_ = VDE + (*E*_HOMO_ – *E*_HOMO–1_)].^74^

**Table 4:** Calculated Relative Energies (*E*_0_), Au^–^ Binding Energies (*D*_0_^–^), VDEs, and First-Excited State Electron Binding Energies (eBE_A_), with Comparison to Experimental Values for All Complexes^a^

				VDE		eBE_A_	
species	complex	*E*_0_	*D*_0_^–^	theor.	exp.	theor.	exp.
Au^–^				2.215	2.308	3.360	3.445
Au^–^[fen]	F1	0	0.548	2.588	2.71 ± 0.05	3.777	3.93 ± 0.02
	F2	0.065	0.483	2.528			
	F3	0.119	0.429	2.490			
	F4	0.128	0.420	2.490			
Au^–^[men]	M1.1	0	0.680	2.669	2.76 ± 0.05	3.905	4.04 ± 0.02
	M1.2	0.186	0.494	2.551			
	M1.3	0.456	0.224	2.326			
Au–[3-HTHF]	T1.1	0	0.761	2.880	3.01 ± 0.03	4.045	4.22 ± 0.01
	T1.2	0.031	0.730	2.860			
	T1.3	0.036	0.725	2.800			
	T1.4	0.268	0.493	2.560			
Au^–^[ala]	A1.1	0	0.736	2.866	3.02 ± 0.03	4.052	4.23 ± 0.01
	A1.2	0.142	0.594	2.702			
	A1.3	0.180	0.557	2.622			
	A2.1	0.010	0.726	2.885			
	A2.2	0.099	0.637	2.863			
	A2.3	0.183	0.554	2.692			
	A3.1	0.069	0.667	2.805			
	A3.2	0.084	0.653	2.869			
	A3.3	0.180	0.557	2.622			

a Energies are given in eV.

For all Au^–^[M] complexes and Au^–^, calculated VDE and eBE_A_ underestimate the corresponding experimental values. For Au^–^, the underestimation of VDE and eBE_A_ is roughly the same (∼0.09 eV). However, all the complexes show greater underestimations in eBE_A_, ranging from 0.16 to 0.18 eV, compared to the underestimations in VDE of the complexes, which ranges from 0.01 to 0.15 eV or 0.13 eV, depending on whether the lowest-energy complex of Au^–^[ala] (A1.1) or the complex with the highest VDE (A2.1) is evaluated. Despite the slight differences shown between theory and experiments, the narrow range of energy difference for our four different Au^–^[M] complexes indicates the suitability of the selected methods for studying these diverse species.

Comparisons can be made between the experimental and predicted frequencies of the OH stretching frequency of Au^–^[3-HTHF] and Au^–^[ala] (see Table 3). Predicted OH stretching frequencies are provided for the anionic (Au^–^[M]) and neutral (Au[M]) complexes and the bare molecule (M). All predicted frequencies for the OH stretch fall within experimental error margins for both Au^–^[3-HTHF] and Au^–^[ala]. It is important to note the predicted redshift in frequency moving from the bare molecule to the anionic complex. For Au^–^[3-HTHF], this shift is calculated to be 536 cm^–1^, and for Au^–^[ala], the shift is 438 cm^–1^. These shifts are indications of H-bound anionic systems, as stated by condition 4 of the IUPAC rules for H bonding.^11^ For Au^–^[ala], the significant redshift is predicted to occur when going from the isolated molecule to the neutral complex, suggesting the possibility for H bonding between neutral Au and ala. However, in Au^–^[3-HTHF], a greater redshift is predicted to occur when going from Au[3-HTHF] to Au^–^[ 3-HTHF], indicating the necessity of the additional charge on Au to form a H bond. Due to the experimental resolution, it is currently not possible to discern whether this redshift occurs in the complex spectra.

### Energy Trends

From Table 4, it is possible to relate the energy *E*_0_ of a complex and its Au^–^ binding energy to the complex’s VDE. In general, the predicted VDE correlates well with *E*_0_, where a lower relative energy equates to greater VDE. This assertion is supported by our experiments as it is expected that experimentally we are most likely to form low-energy isomers, and the experimental VDE of each complex is closest to the highest predicted VDEs, associated with low-energy isomers. However, an exception can be found when comparing A1.1 and A2.1 in Table 4, where the highest VDE does not coincide with the most stable isomer. In Figure 5, the graph of relative energy versus VDE for the Au^–^[men] complexes indicates a somewhat piecewise distribution, where for each binding configuration (i.e., via ring, edge, or isopropyl) the isomers have nearly constant VDE, where the relative energy appears to be partially dictated by the energy of the initial molecular isomer, but the VDEs of the complexes are very close in energy. An exception to this linearity is the isopropyl-binding group, where M4.3 has a much higher VDE and lower *E*_0_ than the other complexes of this group due to the added interaction of the methyl group with Au^–^. For the Au^–^[ala] complexes, a similar analysis can be conducted by analyzing Figure 8, where the complexes are categorized based on the individual contributions by the different H bond donors. For Au^–^[ala], the initial isomer structure plays a lesser role in the relative energy of the complex formed, in comparison to that in Au^–^[men]. Instead, the identity of the primary and secondary H bond donors has the largest effect. By comparing the energies of primary H bond donors, OH and NH, it is clear that a 1′ OH interaction leads to a higher VDE and lower *E*_0_. The weaker secondary binding site (2b′ = NH/CH) does not play a significant role in the VDE (comparing blue and yellow), but the stronger secondary H bond donor does appear to be significant to the VDE as comparing the isomers marked in yellow and green does indicate a clustering of the NH 2a′ H bond donors at higher VDEs compared to the 2a′ CH H bond donors. However, there is no significant difference seen for the spread of relative energies of these two groups.

Of the four different complexes, the complexes without conventional H bond donors, Au^–^[fen] and Au^–^[men], exhibit the smallest experimental VDE shifts and have the lowest predicted values for *D*_0_^–^ and VDE. For Au^–^[HTHF] and Au^–^[ala] that contain a OH group, the energies are predicted and confirmed experimentally to be higher. Overall, the ordering of complexes by increasing experimental VDE is the same as ordering by increasing predicted D_0_^-^, if the isomers with the predicted VDE closest to the experimental VDE are considered.

### Hydrogen Bonding Energetics

Comparing *D*_0_^–^ of the complex of the conventional H bond donor ala with that of the unconventional H bond donor, men, reveals an energetic difference in Au^–^ binding energy of only 0.056 eV. This small separation for these two dissimilar molecules supports not only the importance of identity of the H bond donor in a complex but also the importance of potential cooperativity between many H bond donors in the total H bonding of a complex (*E*_HB_). Additionally, it reveals the different forces that can be at play in forming a hydrogen bond, such as electrostatic forces, induction, or dispersion.^14^

It is possible to first approximate the maximum possible strength of the individual H bonds in the different complexes, by assuming that *D*_0_^–^ = −*E*_HB_, where (*E*_HB_) describes the sum of all the H bonding interactions between the molecule (M) and Au^–^. We have assumed that up to three interactions can significantly contribute to the H bonding of Au^–^ in the considered complexes. Therefore, the maximum energy of a single H bond in any complex must range from *E*_HB_ (assuming only one contributing H bond donor) to *E*_HB_/3 (assuming three equally contributing H bond donors). From this description, the range for the maximum H bond energy for a single H bond for each of the complexes is as follows: Au^–^[fen] = −0.18 to −0.55 eV, Au^–^[men] = −0.23 to −0.68 eV, Au^–^[3-HTHF] = −0.25 to −0.76 eV, and Au^–^[ala] = −0.25 to −0.87 eV. A H bond of moderate strength is indicated by an energy greater than −0.2 eV,^11^ which would indicate that all complexes could contain at least one moderate to strong bonds, by this calculation. Furthermore, considerations can be made for expected individual contributions of the different H bond donors. As the stability of complex structures has been shown to strongly depend on an interaction between OH and Au^–^ in Au^–^[3-HTHF] and Au^–^[ala], it can be assumed that the strongest H bond in these species would most likely have an energy close to the maximum value (*D*_0_^–^) for these complexes. In the case of fen and men, as all H bond donors are the same (i.e., CH) and the bond lengths and angles do not differ significantly, it can be assumed that H bonding in these complexes is closer to the lower limit, where there are three moderate H bonds with approximately the same energy. However, approximating H bond energies in this manner does not account for the other attractive forces that may be present in the complex. Therefore, these estimations should be considered the upper limit on the energy of H bonding in each of the complexes.

To further confirm the existence of H bonding in the four complexes, we consider AIM analysis. The BCPs between Au^–^ and M, the electron density at the determined BCPs (ρ_BCP_), and predictions of the individual H bond energies based on the relation between ρBCP and the binding energy of a single H bond (be_HB_), determined by Emamian et al.,^14^ can be found in Table 5. The AIM analysis identified four BCPs for Au^–^[fen], three BCPs for Au^–^[men], two BCPs for Au^–^[3-HTHF], and two BCPs for Au^–^[ala]. The BCPs identified coincide with the most likely H-bonding functional groups, based on the Au^–^···H bond distances determined from the geometry optimizations. All complexes have ρ_BCP_ values that are on par with those of other known H-bonded complexes (Table 5 of ref (14)). In comparison to specifically the charged H-bonded systems by Emamian et al., the Au^–^[M] complexes do have overall smaller ρ_BCP_.^14^ However, the Au^–^···HO ρ_BCP_ of Au^–^[3-HTHF] and Au^–^[ala] is greater than that of Au^–^···HO ρ_BCP_ (0.026 e/Bohr^3^) found for the experimentally established strong H bonding in the gold(I)···indazol-3-ylidene complex.^20^ Additionally, a second complex studied by Park and Gabbaï et al. with weaker, but still confirmed, gold H bonding possessed a Au^–^···HO ρ_BCP_ of 0.017 e/Bohr^3^.^20^ This value is similar to the greatest Au^–^···HC ρ_BCP_ of Au^–^[men].

**Table 5:** AIM Calculated BCPs, ρ_BCP_ (e/Bohr^3^), and Predicted be_HB_ (eV)

species	BCP	ρ_BCP_	be_HB_^a^
Au^–^[fen]	Au^–^···HC_3_	0.0103	–0.193
	Au^–^···HC_4_	0.0098	–0.186
	Au^–^···HC_5_	0.0067	–0.142
	Au^–^···HC_1_	0.0033	–0.093
Au^–^[men]	Au^–^···HC_4_	0.0156	–0.269
	Au^–^···HC_9_	0.0100	–0.189
	Au^–^···HC_6_	0.0094	–0.180
Au^–^[3-HTHF]	Au^–^···HO_1_	0.0283	–0.450
	Au^–^···HC_4_	0.0066	–0.141
Au^–^[ala]	Au^–^···HO_1_	0.02727	–0.436
	Au^–^···HN_1_	0.0089	–0.173

a be_HB_ are predictions using the relation:  by Emamian et al.^14^

The binding energies of the individual H bonds (be_HB_) have been predicted using the linear relationship established between binding energy and ρ_BCP_ for charged species by Emamian et al.^14^ In comparison to our first approximation of the H-binding energies based on *D*_0_^–^, the case of Au^–^[fen] and Au^–^[men] is relatively well-matched. The spread of energies is fairly similar between the largest BCPs of the complexes, and the average be_HB_ predicted here is similar to that which was shown above for a case where there are three equally contributing H bonds to the total E_HB_ of a complex. For Au^–^[3-HTHF] and Au^–^[ala], there is a larger discrepancy between predictions with AIM descriptors and predictions from *D*_0_^–^. Although the assumption that the Au^–^···HO interaction would contribute the most to *E*_HB_ was correct, the *E*_HB_ determined from summing the predicted be_HB_ from the AIM analysis is much lower than what is assumed above for the two complexes. This discrepancy further supports our claim that predictions of H bond strengths by the assumption of *D*_0_^–^ = −*E*_HB_ will lead to overestimations of the H bond strengths. Therefore, the strengths of the H bonds for the complexes here are re-evaluated and compared to the ranking established by Emamian et al.,^14^ where based on the ρ_BCP_ and be_HB_ values, these complexes are categorized as containing weak- to medium-strength H bonds.

### H Bond Character

**Figure 9:**
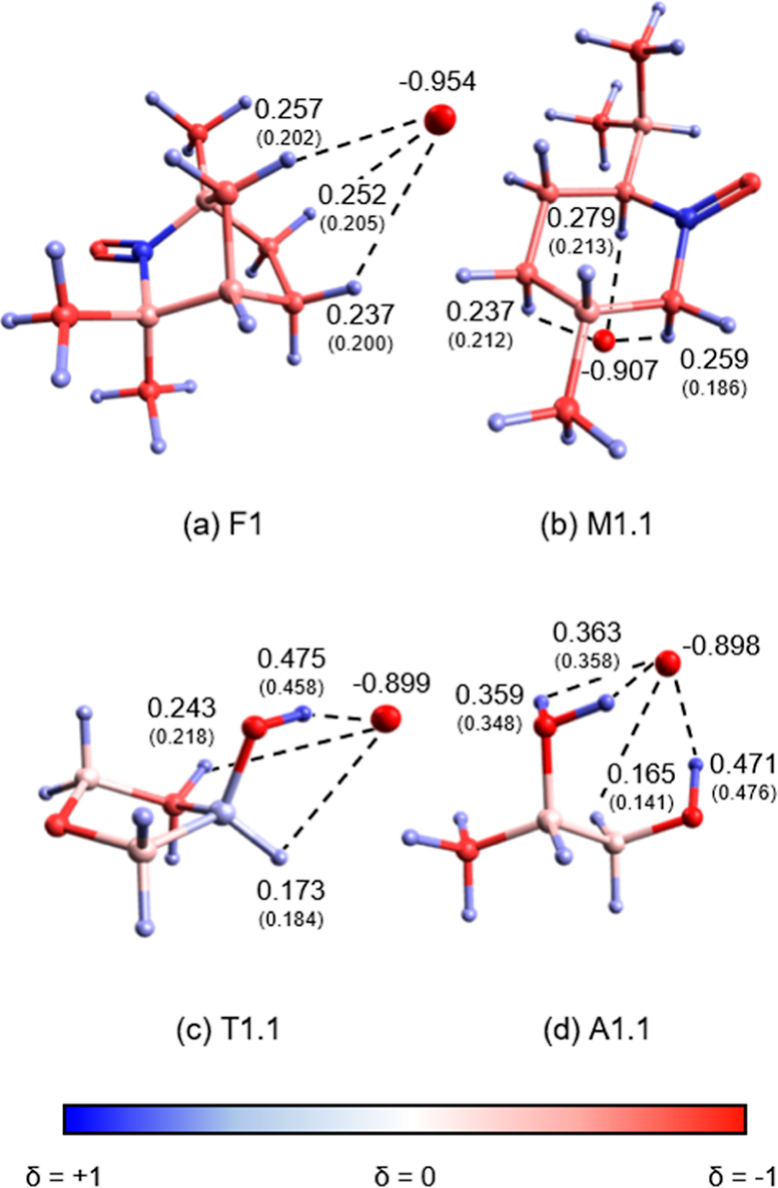
Lowest-energy isomer of Au^–^[fen], Au^–^[men], Au^–^[3-HTHF], and Au^–^[ala] colored by atomic partial charges given in units of *e*. Values in parentheses refer to the isolated molecule.

Emamian et al. have shown that electrostatic and inductive forces are the largest contributors to H bonding in charged complexes.^14^ Induction includes contributions from both polarization and charge transfer. Characterization of the inductive interaction between Au^–^ and the complexing molecule was conducted via an NBO analysis to reveal the localization of the excess charge and the electron transfer from Au^–^ to the molecule. The charge distributions of the most stable complexes F1, M1.1, T1.1, and A1.1 are shown in Figure 9. For each complex, the partial charges of the interacting atoms are given for the anionic complex. For the interacting H atoms, the values in parenthesis are the partial charges of the atoms for the isolated molecule.

In all isomers, the excess charge is primarily located on the gold atom. Comparing the atomic partial charges of Au^–^ and M with those of the complex provides an indication of the charge transfer (Δ*Q*) that occurs. The Au^–^[fen] and Au^–^[men] complexes indicate that smaller charge transfer occurs with values of Δ*Q* = 0.046 and 0.093. For the two alcohols, Au^–^[3-HTHF] and Au^–^[ala], the charge transfer is greater (Au^–^[3-HTHF] Δ*Q* = 0.101 and Au^–^[ala] Δ*Q* = 0.102). With the decrease in negative charge of Au^–^, the H atoms that interact with Au^–^ also indicate an increase in positive partial charge. An exception is the OH hydrogen in ala. This discrepancy is most likely due to the calculation of partial charge of this hydrogen under internal H bonding conditions for the isolated species. This magnitude of charge transfer is expected for H-bonded complexes. Comparing the loss of charge in Au^–^ and gain in charge of the H, it is clear that the gain in charge on the H is much greater in magnitude. This difference indicates an increase in the HX bond polarity due to induction.

We also analyzed the magnitude of the dipole moment of the molecular structure and the angle of the dipole vector with respect to the position of Au^–^ with dependence on *D*_0_^–^ and Δ*Q*. Here, we expected an increase in *D*_0_^–^ and Δ*Q* with increasing magnitude and a decreasing angle of the dipole moment vector because the dipole of the molecule should have an attractive force on Au^–^ if it is strong enough and correctly aligned. However, such a correlation could not be observed. This can be ascribed to the breakdown of the multipole expansion, which is valid at great distances but not here where bond lengths are around 3 Å. Hence, all H bonds emerge from local charge displacements rather than from a global dipole moment.

The NBO analysis of Au^–^[ala] showed significant electron donation from the 6s orbital of Au^–^ into the σ* orbital of the OH bond, with secondary donations into the NH and CH bonds. This behavior was consistent with that of the other anionic complexes, which showed donations into the OH and CH bonds of 3-HTHF and donations into the CH bonds of fen and men, primarily from the 6s orbital. This donation behavior is indicative of a three-center-four-electron bond. Also, primary electron donation from the 6s orbital of Au^–^ could provide some context for the different shifts in the X and A transition binding energies. From the results of the NBO analysis, it can be assumed that the blueshift in the electron binding energy of the X transition upon complexation arises from the sharing of the electrons, leading to a lowering of the HOMO orbital energy. When this occurs, the 6s orbital is expected to become more diffuse. This orbital expansion could lead to deshielding of the 5d orbital, causing lowering of the HOMO – 1 orbital energy, which would, in turn, lead to a blueshift in the A transition. This explanation is supported by the HOMO and HOMO – 1 orbital energies of Au^–^ and Au^–^ [M] (Table S8 in the Supporting Information).

## Conclusions

We have measured the photoelectron spectrum of the auride-bound Au^–^[M] complexes: Au^–^[fen], Au^–^[men], Au^–^[3-HTHF], and Au^–^[ala], and investigated their structures and bonding characteristics with DFT calculations and wavefunction analysis. The experimental results revealed photodetachment transitions that were atomic gold transitions perturbed by attractive interactions with the complexing molecules. The resulting VDEs of the complexes, compared to Au^–^, were blueshifted, and greater VDE shifts were indicative of stronger H bonding between Au^–^ and M. An evaluation of the angular distribution of the photoelectrons for transition X in the complexes supports the perturbative model of photodetachment.

Analyzing the Au^–^[M] structures provided the opportunity to build comparisons of the conventional H bond donors OH and NH with the nonconventional H bond donor CH. Of the H bond donors, OH exhibited the strongest interactions with Au^–^, as evident from the greater elongation of the OH bond upon complexation, the shortest Au^–^···H bond lengths, and the bond angles closest to linearity. This strong interaction resulted in complexes of the two molecules that contained OH functional groups, Au^–^[3-HTHF] and Au^–^[ala], to have larger predicted Au^–^ binding energies and greater experimental VDE shifts. Given the predicted Au^–^ binding energies, the Au^–^···HO bonds can be categorized as moderate H bonds. The only NH H bond donor evaluated played a secondary role, along with the CH H bond donors, to the OH H bond donor in the lowest-energy Au^–^[ala] complexes. These bonds are expected to be weak H bonds. Generally, the complexes Au^–^[fen] and Au^–^[men], which only contained non-conventional H bond donors (CH), had smaller experimental VDE shifts and smaller predicted Au^–^ binding energies, compared to the OH-containing molecules. Also, there was weaker adherence to the geometry conditions of H bonding for the nonconventional H bond donors. However, the calculated charge transfer, AIM analysis, and electron donation character of the Au^–^[fen] and Au^–^[men] complexes mirrored that of the conventionally H-bound complexes Au^–^[3-HTHF] and Au^–^[ala] and are comparable to other well-known H-bonded systems. Therefore, the interaction in Au^–^[fen] and Au^–^[men] can be classified as weak H bonding.

## Supporting Information Available

Anisotropy parameters, complex structures, NH_2_ stretching frequencies, geometric information, additional complex predicted energies, and orbital energies (PDF)
