# Supplementary material for: Comparison of Conventional and Nonconventional Hydrogen Bond Donors in Au– Complexes
Source: J Phys Chem A. 2022 Jun 10;126(24):3880–92. doi: 10.1021/acs.jpca.2c02725 (PMC9234979; doi:10.1021/acs.jpca.2c02725)
Supplement: Supplementary file 1 — jp2c02725_si_001.pdf [file jp2c02725_si_001.pdf]

Supporting Information: A Comparison of Conventional and Non-conventional Hydrogen Bond Donors in Au^- Complexes

Jenny Triptow,[†,‡] Gerard Meijer,[†] André Fielicke,[†,‡] Otto Dopfer,[‡] and Mallory Green[†*]

[†] Fritz-Haber-Institut der Max-Planck Gesellschaft, Faradayweg 4-6, 14195 Berlin, Germany

[‡] Institut für Optik und Atomare Physik, Technische Universität Berlin, Hardenbergstraße 36, 10623 Berlin, Germany

Table S1: Measured anisotropy parameter β for the A transition of the Au^-[M] complexes and Au^-. The corresponding electron kinetic energy eKE is given in eV.

**Species**	**eKE**	**β_A**
**Au^-**	0.688	-0.1 ± 0.05
	0.906	0.0 ± 0.21
**Au^-[fen]**	0.200	0.1 ± 0.04
**Au^-[men]**	0.282	0.1 ± 0.03
**Au^-[3-HTHF]**	0.134	0.1 ± 0.04
**Au^-[ala]**	0.127	0.1 ± 0.05

Table S2: Calculated and scaled NH_2 stretching frequencies for A1, A1.1, and A1.2. A1.1 contains a 1' Au^-···HO interaction and A1.2 contains a 1' Au^-···HN interaction. Frequencies are given in cm^{-1}.

**Conformer**	**symmetric stretch**	**asymmetric stretch**
**A1**	3357	3441
**A1.1**	3310	3380
**A1.2**	3126	3362

Figure S1 Additional complex isomers of Au^-[men].

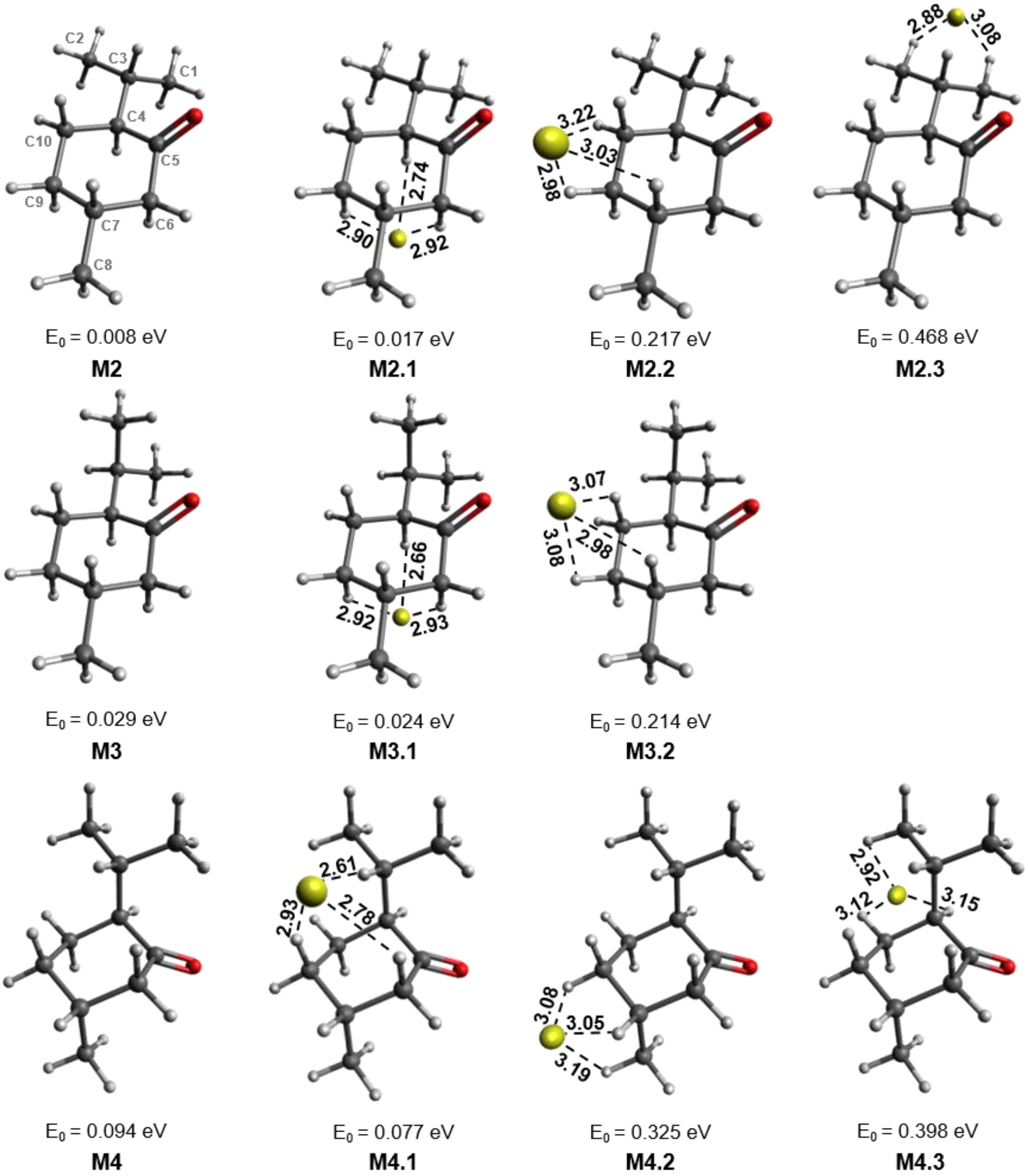

Figure S2: Additional complex isomers of Au^-[3-HTHF].

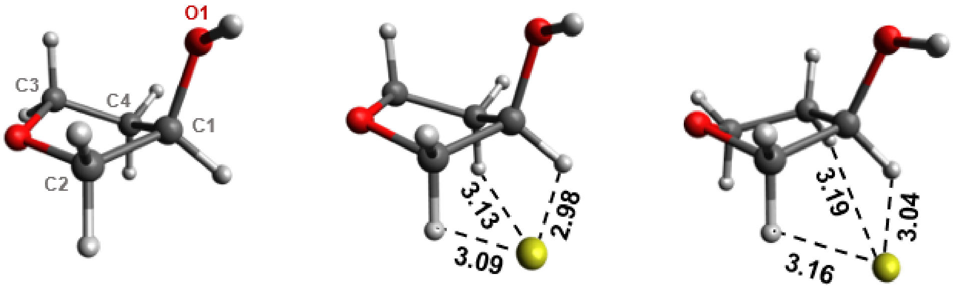

E_0 = 0.012 eV	E_0 = 0.316 eV	E_0 = 0.326 eV
**T2**	**T2.1**	**T2.2**

Figure S3: Additional complex isomers of Au^-[ala].

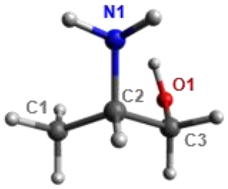

E_0 = 0.24 eV
A2

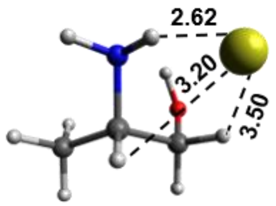

E_0 = 0.183 eV
A2.4

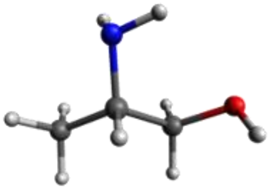

E_0 = 0.69 eV
A4

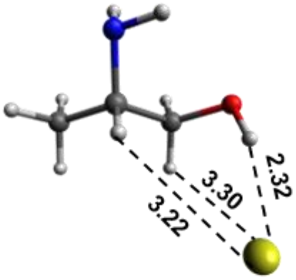

E_0 = 0.08 eV
A4.1

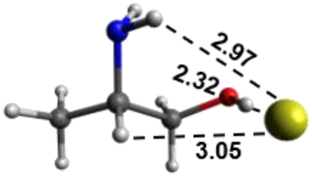

E_0 = 0.082 eV
A4.2

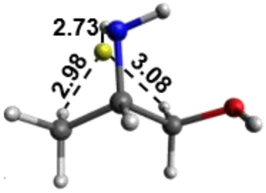

E_0 = 0.360 eV
A4.3

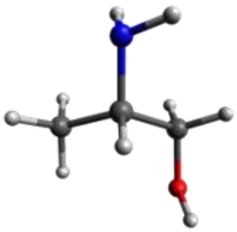

E_0 = 0.101 eV
A5

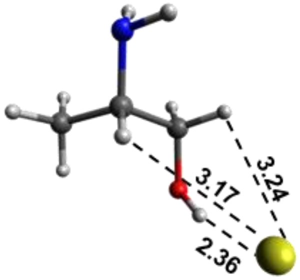

E_0 = 0.112 eV
A5.1

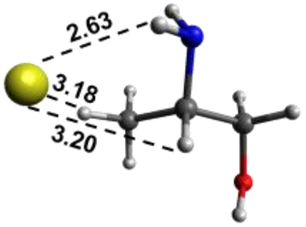

E_0 = 0.332 eV
A5.2

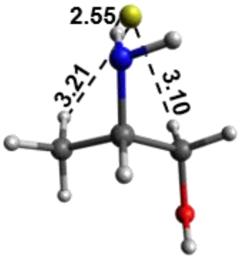

E_0 = 0.354 eV
A5.3

Table S3: Bond lengths and angles for Au^-[fen]. Without Au^- the C-H bond length is 1.09 Å except for C(4,6)-H and one hydrogen of C1 which have a bond length of 1.10 Å.

Complex	H-bond	length C-H [Å]	length H-Au^- [Å]	angle [Å]
**F1**	C3H-Au^-	1.09	2.88	151.2
	C4H-Au^-	1.09	2.93	134.9
	C5H-Au^-	1.09	3.16	128.4
**F2**	C6H-Au^-	1.09	2.92	141.1
	C8H-Au^-	1.09	3.03	145.8
	C9H-Au^-	1.09	3.07	144.4
	C1H-Au^-	1.09	3.21	144.0

**F3**	C3H-Au^-	1.09	2.73	158.5
	C9H-Au^-	1.09	2.97	166.4
**F4**	C4H-Au^-	1.09	3.08	129.0
	C5H-Au^-	1.09	2.94	136.7
	C8H-Au^-	1.09	2.93	168.4

Table S4: Bond lengths and angles for Au^-[men]. Without Au^- the C-H bond length is 1.09 Å except for C(4,7)-H and one hydrogen of C(6,9) which have a bond length of 1.10 Å.

**Complex**	**H-bond**	**length C-H** [Å]	**length H-Au⁻** [Å]	**angle** [°]
**M1.1**	C4H-Au^-	1.11	2.66	161.0
	C6H-Au^-	1.10	2.95	144.3
	C9H-Au^-	1.09	2.91	144.8
**M1.2**	C7H-Au^-	1.10	2.98	117.7
	C9H-Au^-	1.09	3.08	130.7
	C10H-Au^-	1.09	3.07	130.1
**M1.3**	C1H-Au^-	1.09	3.06	151.6
	C2H-Au^-	1.09	2.89	159.5
	C3H-Au^-	1.09	4.27	87.9

Table S5: Bond lengths and angles for Au^{-}[3HTHF]. Without Au^{-} the C-H bond length is 1.09 Å, except for one hydrogen of C2 which has a length of 1.10 Å. The O-H bond length is 0.96 Å for the bare molecule.

**Complex**	**H-bond**	**length (C,O)-H [Å]**	**length H-Au⁻ [Å]**	**angle [°]**
**T1.1**	C1H-Au^{-}	1.09	3.50	92.2
	C4H-Au^{-}	1.09	3.18	124.8
	OH-Au^{-}	0.99	2.13	164.6
**T1.2**	C1H-Au^{-}	1.09	3.37	96.3
	C2H-Au^{-}	1.09	3.39	111.2
	OH-Au^{-}	0.99	2.31	162.1
**T1.3**	C2H-Au^{-}	1.10	3.21	127.2
	C3H-Au^{-}	1.10	3.12	128.6
	C4H-Au^{-}	1.09	3.19	115.5
	OH-Au^{-}	0.99	2.34	169.0
**T1.4**	C2H-Au^{-}	1.10	2.99	139.5
	C3H-Au^{-}	1.09	3.25	121.9
	C4H-Au^{-}	1.09	2.92	131.1

Table S6: Bond lengths and angles for Au^-[Ala]. Without Au^- the bond length of C-H is 1.09 Å, except for one hydrogen of C3, which has a bond length of 1.10 Å. N-H shows a bond length of 1.01 Å and O-H has a length of 0.96 Å for the bare molecule. O-H has a bond length of 0.97 Å for A1.

Complex	bond	length (C,O,N)-H [Å]	length H-Au⁻ [Å]	angle [°]
**A1.1**	C3H-Au^-	1.10	3.23	107.1
	NH-Au^-	1.02	3.31	102.0
	NH-Au^-	1.02	3.01	122.4
	OH-Au^-	0.99	2.33	163.5
**A1.2**	C1H-Au^-	1.09	3.09	136.2
	C2H-Au^-	1.09	3.66	94.8
	NH-Au^-	1.03	2.51	157.9
**A1.3**	C1H-Au^-	1.09	3.01	140.3
	C3H-Au^-	1.10	3.43	131.6
	NH-Au^-	1.03	2.52	167.3
**A2.1**	C3H-Au^-	1.10	3.24	107.0
	NH-Au^-	1.02	3.00	122.6
	OH-Au^-	0.99	2.33	163.9
**A2.2**	C1H-Au^-	1.09	3.73	126.8
	NH-Au^-	1.03	2.53	171.6
	OH-Au^-	0.99	2.32	173.5
**A2.3**	C1H-Au^-	1.09	3.14	134.5
	C2H-Au^-	1.10	3.63	95.2
	NH-Au^-	1.03	2.53	150.8
**A3.1**	C2H-Au^-	1.09	3.07	127.6
	C3H-Au^-	1.10	3.50	99.9
	OH-Au^-	0.99	2.32	162.2
**A3.2**	C2H-Au^-	1.09	3.90	121.1
	NH-Au^-	1.02	2.54	138.5
	OH-Au^-	0.99	2.32	170.0

Table S7: Calculated relative energies (E_0), Au^- binding energies (D_0^-), and vertical detachment energies (VDE) for the additional complex isomers. Energies are given in eV.

Species	Complex	E_0	D_0^-	VDE
**Au^-**	--	--	--	2.215
**Au^-[men]**	M2.1	0.017	0.663	2.660
	M2.2	0.217	0.463	2.544
	M2.3	0.468	0.212	2.325
	M3.1	0.024	0.656	2.674
	M3.2	0.214	0.466	2.554
	M4.1	0.077	0.603	2.698
	M4.2	0.325	0.355	2.543
	M4.3	0.398	0.282	2.439
**Au^-[3-HTHF]**	T2.1	0.316	0.445	2.548
	T2.2	0.326	0.435	2.539
**Au^-[ala]**	A2.4	0.183	0.553	2.688
	A4.1	0.069	0.667	2.805
	A4.2	0.084	0.653	2.869
	A4.1	0.079	0.657	2.823
	A4.2	0.082	0.655	2.855
	A4.3	0.360	0.376	2.502
	A5.1	0.112	0.625	2.837
	A5.2	0.332	0.404	2.639
	A5.3	0.354	0.382	2.532

Table S8: Calculated HOMO and HOMO-1 orbital energies and orbital shifts from gold anion detachment, given in eV for Au^- and the most stable Au^-[M] complexes.

Species	HOMO	HOMO-1	Δ_{HOMO}	Δ_{HOMO-1}
**Au^-**	0.326	-0.750	--	--
**F1**	-0.582	-1.711	-0.908	-1.021
**M1.1**	-0.719	-1.955	-1.045	-1.205
**T1.1**	-0.765	-1.930	-1.091	-1.180

**A1.1**	-0.804	-1.990	-1.130	-1.240
